# Health-related quality of life in lower-risk MDS patients compared with age- and sex-matched reference populations: a European LeukemiaNet study

**DOI:** 10.1038/s41375-018-0089-x

**Published:** 2018-03-06

**Authors:** Reinhard Stauder, Ge Yu, Karin A. Koinig, Tim Bagguley, Pierre Fenaux, Argiris Symeonidis, Guillermo Sanz, Jaroslav Cermak, Moshe Mittelman, Eva Hellström-Lindberg, Saskia Langemeijer, Mette Skov Holm, Krzysztof Mądry, Luca Malcovati, Aurelia Tatic, Ulrich Germing, Aleksandar Savic, Corine van Marrewijk, Agnès Guerci-Bresler, Elisa Luño, Jackie Droste, Fabio Efficace, Alex Smith, David Bowen, Theo de Witte

**Affiliations:** 1 Department of Internal Medicine V (Hematology and Oncology), Medical University Innsbruck, Innsbruck, Austria; 2 Epidemiology and Cancer Statistics Group, Department of Health Sciences, University of York, New York, United Kingdom; 3 Service d’Hématologie, Hôpital Saint-Louis, Assistance Publique des Hôpitaux de Paris (AP-HP) and Université Paris 7, Paris, France; 4 Department of Medicine, Division of Hematology, University of Patras Medical School, Patras, Greece; 5 Department of Hematology, Hospital Universitario y Politécnico La Fe, Valencia, Spain; 6 CIBERONC, Instituto Carlos III, Madrid, Spain; 7 Department of Clinical Hematology, Institute of Hematology & Blood Transfusion, Praha, Czech Republic; 8 Department of Medicine A, Tel Aviv Sourasky (Ichilov) Medical Center and Sackler Medical Faculty, Tel Aviv University, Tel Aviv, Israel; 9 Department of Medicine, Division of Hematology, Karolinska Institutet, Stockholm, Sweden; 10 Department of Hematology, Radboud University Medical Center, Nijmegen, Netherlands; 11 Department of Hematology, Aarhus University Hospital, Aarhus, Denmark; 12 Department of Hematology, Oncology and Internal Medicine, Warszawa Medical University, Warszawa, Poland; 13 Department of Hematology Oncology, Fondazione IRCCS Policlinico San Matteo, University of Pavia, Pavia, Italy; 14 Center of Hematology and Bone Marrow Transplantation, Fundeni Clinical Institute, Bucharest, Romania; 15 Department of Hematology, Oncology and Clinical Immunology, Universitätsklinik Düsseldorf, Düsseldorf, Germany; 16 Clinic of Hematology - Clinical Center of Vojvodina, University of Novi Sad, Novi Sad, Serbia; 17 Service d’Hématologie, Center Hospitalier Universitaire Brabois Vandoeuvre, Nancy, France; 18 Servicio d’Hematología, Servicio de Salud del Principado de Asturias Oviedo, Oviedo, Spain; 19 Fondazione GIMEMA Onlus, Rome, Italy; 20 St. James’s Institute of Oncology, Leeds Teaching Hospitals, Leeds, United Kingdom; 21 Department of Tumor Immunology - Nijmegen Center for Molecular Life Sciences, Radboud University Medical Center, Nijmegen, Netherlands

## Abstract

In myelodysplastic syndromes (MDS), health-related quality of life (HRQoL) represents a relevant patient-reported outcome, which is essential in individualized therapy planning. Prospective data on HRQoL in lower-risk MDS remain rare. We assessed HRQOL by EQ-5D questionnaire at initial diagnosis in 1690 consecutive IPSS-Low/Int-1 MDS patients from the European LeukemiaNet Registry. Impairments were compared with age- and sex-matched EuroQol Group norms. A significant proportion of MDS patients reported moderate/severe problems in the dimensions pain/discomfort (49.5%), mobility (41.0%), anxiety/depression (37.9%), and usual activities (36.1%). Limitations in mobility, self-care, usual activities, pain/discomfort, and EQ-VAS were significantly more frequent in the old, in females, and in those with high co-morbidity burden, low haemoglobin levels, or red blood cells transfusion need (*p* < 0.001). In comparison to age- and sex-matched peers, the proportion of problems in usual activities and anxiety/depression was significantly higher in MDS patients (*p* < 0.001). MDS-related restrictions in the dimension mobility were most prominent in males, and in older people (*p* < 0.001); in anxiety/depression in females and in younger people (*p* < 0.001); and in EQ-VAS in women and in persons older than 75 years (*p* < 0.05). Patients newly diagnosed with IPSS lower-risk MDS experience a pronounced reduction in HRQoL and a clustering of restrictions in distinct dimensions of HRQoL as compared with reference populations.

## Introduction

Myelodysplastic syndromes (MDS) represent challenging hematopoietic disorders characterized by cytopenias, functional blood defects, and clonal hematopoiesis. The clinical course is characterized by an impaired health-related quality of life (HRQoL), the risk of transformation to acute myeloid leukaemia (AML) and reduced survival in the majority of patients [1]. Based on biological parameters, the patients are classified into different risk groups to predict overall survival (OS) and the risk of AML transformation. The international prognostic scoring system (IPSS) [2] and more recently, the revised IPSS (IPSS-R) [3] represent the gold standard in prognostication of MDS. Based on these scoring systems, IPSS low/intermediate-1 risk and IPSS-R (very) low/intermediate risk are classified as lower-risk MDS with a low propensity to transform to AML [2, 3]. The treatment goals in this cohort of patients are an improvement in cytopenias, prolongation of survival, and improvement and maintenance of HRQoL and functional capacities. IPSS intermediate-2/high and IPSS-R high/very high risk are classified as higher-risk MDS, which are characterized by an increased risk of AML transformation and a short median survival of less than 2 years [1].

Patients with MDS often suffer from a high symptom burden, resulting in restrictions in HRQoL. Assesssment of HRQoL provides information on the patient´s perspective and perception, thus representing a relevant patient-reported outcome (PRO) [1, 4, 5]. The study of HRQoL has become an increasingly critical area of research [6], as limitations in HRQoL are frequently observed in MDS and are only partially explained by anaemia [7, 8]. Moreover, restrictions in HRQoL may predict an unfavourable clinical outcome [9–12]. In addition HRQoL represents a parameter of response evaluation [1, 13, 14]. Thus, the integration of assessment of HRQoL in MDS has been propagated by clinicians, stakeholders, and authorities [1, 13–15]. However, definitive data on HRQoL in low-risk MDS at initial diagnosis are limited by small sample size [16, 17], selection bias [7, 16, 17], and assessment later after initial diagnosis [7, 11, 16, 18, 19]. In addition, most studies have included patients with higher-risk MDS [9–12, 16, 18–20], AML [10, 11], or CMML [11, 16], which precludes precise interpretation. Lower-risk patients with MDS are typically of advanced age with a median of 74 years at diagnosis [21]. The dissection between age-associated restrictions in HRQoL and the incremental impact of MDS in these patients is relevant, yet has not been analyzed at all.

The main objective of this international prospective cohort observational study is to investigate the HRQoL profile of patients with lower-risk MDS at the time of diagnosis, as compared with the general population matched on age and sex. The incremental impact of MDS on symptom burden is dissected by comparing features in MDS with the general population. A secondary objective is to examine clinical factors associated with HRQoL of these patients.

## Materials/methods

### Participants

The EUMDS Registry is a prospective, non-interventional longitudinal study, enroling newly diagnosed patients with IPSS low or intermediate-1 MDS from 145 haematology centres in 17 European countries and Israel. Patients with an IPSS risk intermediate-2 or high, or with therapy-related MDS were excluded. Patients without cytogenetic information were only included if the diagnosis of MDS was morphologically proven, with <5% bone marrow blasts and at most a single cytopenia according to the IPSS. Based on these criteria, exclusively IPPS low or intermediate-1 patients were included in EUMDS.

Therapy is given according to local guidelines [21]. Enrolment was within 100 days of the diagnostic bone marrow aspirate. The average time from date of diagnosis to inclusion was 44 days (standard deviation 28 days). Details on design and data collection have been published elsewhere [21].

As the European Quality of Life five Dimensions (EQ-5D) was not licensed in two countries, 15 countries were included in this analysis. EUMDS (ClinicalTrials.gov: NCT00600860) has been approved by the ethics committees of all participating centres and is performed in accordance with the Declaration of Helsinki. Written informed consent was obtained from all patients.

### HRQoL measurement

Patient-reported HRQoL was measured by EQ-5D, at the time of study enrolment. EQ-5D is a validated, generic, HRQoL questionnaire [22], consisting of the EQ-5D descriptive system with five dimensions related to daily activities (mobility, self-care, usual activities, pain/discomfort, anxiety/depression), with three-level answers (no problem, some problems, severe problems), and a visual analogue scale (EQ-VAS). The five dimensions were converted into a single summary index (EQ-5D index) by applying the European value set (EVS) [23]. EQ-VAS [22] is a global evaluation of ‘own health today’ using a health state scale ranging from 0 (worst imaginable) to 100 (best imaginable).

### Measures of population norms

The main objective of this paper was to compare the QoL of patients with MDS with general population with a similar age and gender distribution. Therefore, population norms were used as reference values to assess the relative HRQoL of patients in comparison to that of an average person [24]. Population norms are based on descriptions of current health status from population surveys. Nine European countries in this study (Denmark, France, Germany, Greece, Italy, Netherlands, Spain, Sweden, and the UK) have reported a series of tables of age/sex population norms for the EQ-5D for both, profile data and VAS scores [25]. For the five European countries and Israel for which there are no published EQ-5D population norms, we replaced the missing data on the probabilities of being in a given level for each EQ-5D dimension with the mean of the available European countries by matching the combination of age group and gender.

### Demographic and clinical parameters

Information on patients’ demographics (age and gender), IPSS-R, co-morbidity index (MDS-CI), haemoglobin (Hb) level at the time of diagnosis, and red blood cell transfusions (RBCT) in the year prior to the diagnosis were recorded [3, 21, 26]. Due to the small number of young adult patients, age was categorized into three groups (<60, 60–75, and 75+ years) to compare HRQoL of different age groups.

### Statistical analysis

Differences in response between the five EQ-5D dimensions in patients with MDS and European norms were evaluated using *χ*^2^ tests. For both EQ-5D index and EQ-VAS, the mean score with standard deviation was calculated. Wilcoxon’s signed ranks tests were conducted to identify any major difference between the MDS patient baseline values and European norms. The relationship between HRQoL and demographic/clinical factors was examined using multilevel linear regression (additional information is available in Supplementary Materials); univariate analysis was performed for age at diagnosis, gender, IPSS-R, MDS-CI, Hb, and RBCT status, and a multivariate analysis was performed adjusting for all other variables. We assessed the discriminative ability of HRQoL not only by a significant difference, but also by a minimally important difference (MID) [27]. The MID is viewed as the smallest difference in score in the domain of interest that is perceived by patients as beneficial or that would result in a change in treatment. See Supplementary Materials for more detail.

All analyses were undertaken in Stata 14 (StataCorp, College Station, TX).

## Results

### Characteristics of patients

Based on IPSS-scoring, i.e., the gold-standard in classification at the time of start of the registry, 1985 patients were included between December 2007 and January 2016, among which 961 (48.4%) were IPSS low-risk and 912 (45.9%) were IPSS Int-1. IPSS score could not be calculated in 5.6% of patients where cytogenetic testing was not available or had failed. Based on inclusion criteria, exclusively IPSS low or int-1 patients were included. Retrospective classification by IPSS-R revealed a (very) low risk in 24.8% and 37.6%, an intermediate risk in 21.2%, high/very high risk in 6.1%, and classification was unknown in 10.3% of patients. In total, 1690 patients (85.1%) completed both EQ-5D descriptive system and EQ-VAS. Thirty-three patients (1.7%) completed EQ-5D description only, and seven patients (0.3%) completed EQ-VAS only (Table 1.). The majority of patients had advanced age (median age: 74 years), and a male preponderance was observed. Nearly half of patients were characterized by Hb levels <10 g/dL at baseline, and more than 30% of patients had received RBCT within 1 year prior to diagnosis. Demographic characteristics of the patients who completed EQ-5D did not differ substantially from the total cohort, showing a similar age distribution and a slightly higher proportion of men. Overall, the HRQoL data in our sample were likely missing at random (Table 1).

**Table 1:** Demographic and clinical characteristics of MDS-patients—entire cohort and EQ-5D respondents

	Total		EQ-5D Completed^a^		EQ-5D not completed	
Characteristic	No. of Patients	%	No. of patients	%	No. of patients	%
Entire cohort	1 985	100.0	1 690	85.1	295	14.9
Age, years						
<60	214	10.8	187	11.1	27	9.2
60–75	818	41.2	707	41.8	111	37.6
75+	953	48.0	796	47.1	157	53.2
Gender						
Male	1 202	60.6	1 039	61.5	163	55.3
Female	783	39.4	651	38.5	132	44.7
Diagnosis (WHO 2001)						
RA	355	17.9	283	16.7	72	24.4
RARS	310	15.6	276	16.3	34	11.5
RCMD	755	38.0	651	38.5	104	35.3
RCMD-RS	118	5.9	102	6.0	16	5.4
RAEB-1	239	12.0	207	12.2	32	10.8
RAEB-2	9	0.5	8	0.5	1	0.3
MDS-U	81	4.1	68	4.0	13	4.4
5q-Syndrome	118	5.9	95	5.6	23	7.8
IPSS						
Low risk	961	48.4	813	48.1	148	50.3
Intermediate-1	912	45.9	782	46.3	130	43.9
Low/int-1 no cytogenetics^b^	112	5.6	95	5.6	17	5.7
IPSS-R						
Very low risk	493	24.8	433	25.6	60	20.3
Low risk	746	37.6	646	38.2	100	33.9
Intermediate risk	420	21.2	341	20.2	79	26.8
High/very high risk	121	6.1	110	6.5	11	3.7
Unknown	205	10.3	160	9.5	45	15.3
MDS-CI						
Low risk	1 276	64.3	1 076	63.7	200	67.8
Intermediate risk	606	30.5	525	31.1	81	27.5
High risk	103	5.2	89	5.3	14	4.7
Haemoglobin (g/dL)						
≥10	1 076	54.2	913	54.0	163	55.3
<10	884	44.5	768	45.4	116	39.3
Unknown	25	1.3	9	0.5	16	5.4
Red blood cell transfusion^c^						
No	1 390	70.0	1 163	68.8	227	76.9
Yes	595	30.0	527	31.2	68	23.1

*WHO* World Health Organization, *IPSS* International Prognostic Scoring System, *IPSS-R* Revised International Prognostic Scoring System, *MDS-CI* Myelodysplastic Syndrome-Comorbidity Index, *HCT-CI* Hematopoietic Cell Transplant-Comorbidity Index

^a^ Includes EQ-5D completed only, EQ-VAS completed only, and both completed

^b^ Patients with cytogenetics failed or not available were included if the diagnosis of MDS was morphologically proven, with <5% bone marrow blasts and at most a single cytopenia according to the IPSS. Based on these criteria, exclusively IPPS low or int-1 patients were included in this cohort

^c^ As assessed in the year prior to initial diagnosis

### Patients with MDS reveal profound impairments in HRQoL

The MDS cohort was characterized by a mean EQ-5D index-score of 0.74 and a mean EQ-VAS of 69.6. A significant proportion of MDS patients reported moderate or severe problems in the dimensions pain/discomfort (49.5%), mobility (41.0%), anxiety/depression (37.9%), and usual activities (36.1%), respectively. The dimension with the lowest proportion of restrictions was self-care (13.3%) (Table 2). Clinically meaningful restrictions in the dimensions mobility, self-care, usual activities, and pain/discomfort as well as in EQ-VAS and EQ-5D index were observed significantly more often in older patients and in those with a high co-morbidity burden, low Hb-levels, or RBCT need (*p* < 0.001). Increased problems with anxiety/depression were significantly more frequent in women (*p* < 0.001) and in patients with lower Hb-levels (*p* < 0.01). The impact of both of IPSS and IPSS-R on EQ-5D scoring was only marginal. In general, restrictions in all parameters of EQ-5D were significantly more often reported in female patients (*p* < 0.05, Table 2).

**Table 2:** Prevalence of problems in distinct dimensions of EQ-5D, EQ-5D index, and EQ-VAS in MDS patients

	**Mobility** problem^a^		**Self-care** problem^a^		**Usual activities** problem^a^		**Pain/discomfort** problem^a^		**Anxiety/depression** problem^a^		**EQ-5D: index**				**EQ-5D: VAS**			
	%	*p*	%	*p*	%	*p*	%	*p*	%	*p*	Mean	SD	*N*	*p*	Mean	SD	*N*	*p*
Total	41.0		13.3		36.1		49.5		37.9		0.74	0.23	1683		69.6	20.1	1657	
Sex		**0.007**		**0.030**		**0.021**		**<0.001**		**<0.001**				**<0.001**				**0.005**
Male	39.1		11.6		33.6		45.5		30.1		0.77	0.22	1035		70.70	20.02	1022	
Female	44.0		16.0		40.0		55.9		50.3		0.69	0.23	648		67.83	20.19	635	
Age group (years)		**<0.001**		**<0.001**		**<0.001**		**<0.001**		0.581				**<0.001**				**<0.001**
<60	18.5		2.7		26.6		31.5		40.8		0.80	0.22	184		76.65	19.35	185	
60–75	33.0		8.5		29.1		43.5		35.9		0.78	0.21	705		72.72	19.95	694	
75+	53.3		20.0		44.5		58.9		39.0		0.69	0.23	794		65.14	19.48	778	
IPSS		0.083		0.057		0.899		**0.005**		0.884				0.845				0.298
Low risk	42.4		13.2		49.6		53.1		39.2		0.74	0.23	809		70.20	19.84	798	
Intermediate risk	38.2		13.1		47.7		49.3		36.8		0.75	0.22	780		68.98	20.27	764	
Low/int-1 no cytogenetics^b^	51.6		16.1		64.5		44.7		36.6		0.70	0.23	93		69.34	21.40	94	
IPSS-R		0.656		0.907		0.899		**0.023**		0.119				0.769				**0.044**
Very low risk	40.6		11.9		32.4		53.1		33.8		0.75	0.23	429		71.40	19.46	422	
Low risk	40.8		13.0		36.6		49.3		38.9		0.73	0.23	645		69.05	20.38	637	
Intermediate risk	42.4		14.4		36.5		44.7		42.9		0.73	0.22	340		68.29	20.76	333	
High/very high risk	40.4		14.7		35.8		52.3		36.7		0.76	0.21	109		68.61	21.16	109	
Unknown	40.6		15.0		43.1		48.8		35.0		0.74	0.22	160		70.49	18.57	156	
MDS-CI		**<0.001**		**<0.001**		**<0.001**		**<0.001**		0.493				**<0.001**				**<0.001**
Low risk	33.9		10.1		31.6		44.5		37.3		0.76	0.22	1072		72.59	19.38	1053	
Intermediate risk	51.8		18.4		42.8		57.2		38.4		0.70	0.23	523		64.92	20.09	518	
High risk	63.6		22.7		50.0		64.8		42.0		0.67	0.25	88		61.26	21.76	86	
Haemoglobin (g/dL)		**<0.001**		**<0.001**		**<0.001**		**0.026**		**0.002**				**<0.001**				**<0.001**
≥10	34.5		9.2		28.9		46.9		34.3		0.77	0.22	909		72.71	19.44	893	
<10	49.2		18.3		45.0		53.2		42.4		0.70	0.23	765		65.79	20.31	755	
Unknown	0.0		0.0		0.0		0.0		22.2		0.95	0.10	9		80.56	15.30	9	
Red blood cell transfusion^c^		**<0.001**		**<0.001**		**<0.001**		**0.049**		0.070				**<0.001**				**<0.001**
No	35.9		9.8		30.9		47.5		36.2		0.76	0.22	1160		71.74	19.56	1137	
Yes	52.2		21.0		47.4		53.9		41.7		0.69	0.24	523		64.94	20.57	520	

*IPSS-R* Revised International Prognostic Scoring System, *MDS-CI* myelodysplastic syndrome-comorbidity index. Bold numbers emphasize significant differences (p<0.05).

^a^ Problem: moderate or severe problems

^b^ Patients with cytogenetics failed or not available were included if the diagnosis of MDS was morphologically proven, with <5% bone marrow blasts and at most a single cytopenia according to the IPSS. Based on these criteria, exclusively IPPS low or int-1 patients were included in EUMDS

^c^ As assessed in the year prior to initial diagnosis

### Association of restrictions in HRQoL and demographic and disease factors

To assess possible associations between clinical parameters and HRQoL, univariate and multivariate linear analyses were performed. It was estimated that patients in the reference group of each of demographic and clinical parameters would have a mean score of 0.85 on the EQ-5D index, and 80.85 on the EQ-VAS (Table 3). Relative to these scores, there was a significant loss in HRQL for groups who were older (e.g., 75+ vs. <60 years; index: −0.08; VAS: −7.33), female, or had increased comorbidities, low Hb-levels, or transfusion dependence (Table 3). These differences exceeded the MID on each of the two HRQL measures (>0.03 on the EQ-5D index and >3.0 on the EQ-VAS). In summary, HRQoL as defined by EQ-5D index and EQ-VAS was more often significantly impaired in older and in female patients and in persons with advanced comorbidities, low Hb levels, and increased transfusion need both in uni- and in multivariate analyses.

**Table 3:** Association of HRQL and demographic and disease characteristics in MDS patients based on univariate and multivariate multilevel linear regression analyses

	EQ-5D index (*n* = 1683 patients)								EQ-VAS (*n* = 1657 patients)							
	Univariate				Multivariate^a^				Univariate				Multivariate^a^			
Variable	Coef.	95% CI		*p*	Coef.	95% CI		*p*	Coef.	95% CI		*p*	Coef.	95% CI		*p*
Constant	0.74	0.73	0.76	**<0.001**	0.85	0.81	0.89	**<0.001**	70.71	67.98	73.44	**<0.001**	80.85	76.88	84.82	**<0.001**
Age group																
<60																
60–75	−0.03	−0.06	0.01	0.144	−0.02	−0.05	0.02	0.287	−3.12	−6.23	−0.01	0.050	−1.85	−4.88	1.18	0.231
75+	−0.11	−0.14	−0.07	**<0.001**	−0.08	−0.12	−0.05	**<0.001**	−10.06	−13.19	−6.93	**<0.001**	−7.33	−10.42	−4.24	**<0.001**
Sex																
Male																
Female	−0.07	−0.09	−0.05	**<0.001**	−0.08	−0.10	−0.06	**<0.001**	−3.24	−5.17	−1.32	**<0.001**	−3.65	−5.51	−1.78	**<0.001**
IPSS-R																
Very low risk																
Low risk	−0.01	−0.04	0.02	0.414	0.03	0.00	0.06	**0.045**	−2.19	−4.59	0.21	0.073	1.41	−1.04	3.87	0.260
Intermediate/high risk	−0.01	−0.04	0.03	0.750	0.04	0.01	0.07	**0.022**	−2.68	−5.42	0.06	0.055	1.62	−1.28	4.52	0.274
Unknown	0.00	−0.04	0.04	0.909	0.02	−0.02	0.07	0.254	−0.01	−3.69	3.67	0.997	3.12	−0.55	6.79	0.095
MDS-CI																
Low risk																
Intermediate/high risk	−0.07	−0.09	−0.04	**<0.001**	−0.06	−0.08	−0.04	**<0.001**	−7.33	−9.26	−5.39	**<0.001**	−6.22	−8.15	−4.28	**<0.001**
Haemoglobin (g/dL)																
≥10																
<10	−0.07	−0.09	−0.04	**<0.001**	−0.05	−0.08	−0.03	**<0.001**	−7.12	−8.99	−5.24	**<0.001**	−5.56	−7.77	−3.35	**<0.001**
Red blood cell transfusion^b^																
No																
Yes	−0.07	−0.10	−0.05	**<0.001**	−0.04	−0.07	−0.02	**<0.001**	−7.14	−9.14	−5.13	**<0.001**	−4.03	−6.18	−1.87	**<0.001**

*IPSS-R* Revised International Prognostic Scoring System, *MDS-CI* Myelodysplastic Syndrome-Comorbidity Index syndrome-comorbidity index. Bold numbers emphasize significant differences (p<0.05).

^a^ Adjusted for all other variables

^b^ As assessed in the year prior to initial diagnosis

### Comparison of HRQoL in MDS and in age- and sex-matched reference populations

We compared subgroups of MDS patients with age- and sex-matched reference norms. Overall, patients with MDS were characterized by a small, but significantly lower EQ-5D index (0.74 vs. 0.76) and lower EQ-VAS (69.6 vs. 71.8) than European norms (*p* < 0.05) (Table 4). However, these differences were too small to fulfil the criteria of MID. In contrast, distinct differences which fulfilled the criteria of a MID were seen in individual components of EQ-5D: a significantly higher proportion of MDS patients reported moderate/severe problems in the dimensions mobility, usual activities, and anxiety/depression compared to the reference populations (*p* < 0.001) (Table 4).

**Table 4:** Comparison of HRQL in MDS patients and age- and sex-matched European reference cohorts

	Mobility problem^a^		Self-care problem^a^		Usual activities problem^a^		Pain/discomfort problem^a^		Anxiety/depression problem^a^		EQ-5D: index				EQ-5D: VAS			
	%	*p*	%	*p*	%	*p*	%	*p*	%	*p*	Mean	SD	*N*	*p*	Mean	SD	*N*	*p*
Entire cohort		**<0.001**		0.438		**<0.001**		0.919		**<0.001**				**0.019**				**0.029**
European Norm	33.5		12.4		26.0		48.8		14.9		0.76	0.18	1683		71.8	3.1	1657	
EUMDS	41.0		13.3		36.1		49.5		37.9		0.74	0.23	1683		69.6	20.1	1657	
Male		**<0.001**		0.409		**<0.001**		0.371		**<0.001**				0.059				0.268
European Norm	29.4		10.7		23.4		43.9		13.7		0.79	0.16	1035		72.6	2.8	1022	
EUMDS	39.1		11.6		33.6		45.5		30.1		0.77	0.22	1035		70.7	20.0	1022	
Female		0.142		0.820		**<0.001**		0.355		**<0.001**				0.164				**0.039**
European Norm	40.0		15.0		30.1		56.8		16.7		0.72	0.19	648		70.5	3.1	635	
EUMDS	44.0		16.0		40.0		55.9		50.3		0.69	0.23	648		67.8	20.2	635	
Age group, <60		0.202		0.288		**<0.001**		0.645		**<0.001**				**0.019**				0.508
European Norm	13.6		4.9		11.4		28.3		9.8		0.86	0.15	184		77.3	2.9	185	
EUMDS	18.5		2.7		26.6		31.5		40.8		0.80	0.22	184		76.7	19.3	185	
Age group, 60–75		**0.002**		0.179		**<0.001**		0.606		**<0.001**				0.261				0.086
European Norm	25.4		6.7		20.0		44.5		14.9		0.79	0.17	705		73.1	1.7	694	
EUMDS	33.0		8.5		29.1		43.5		35.9		0.78	0.21	705		72.7	20.0	694	
Age group, 75+		**<0.001**		0.711		**<0.001**		0.671		**<0.001**				0.207				**<0.001**
European Norm	45.2		19.1		34.6		57.4		16.0		0.71	0.17	794		69.3	1.1	778	
EUMDS	53.3		20.0		44.5		58.9		39.0		0.69	0.23	794		65.1	19.5	778	

^a^ Problem: moderate or severe problems. Bold numbers emphasize significant differences (p<0.05).

**Fig. 1:**
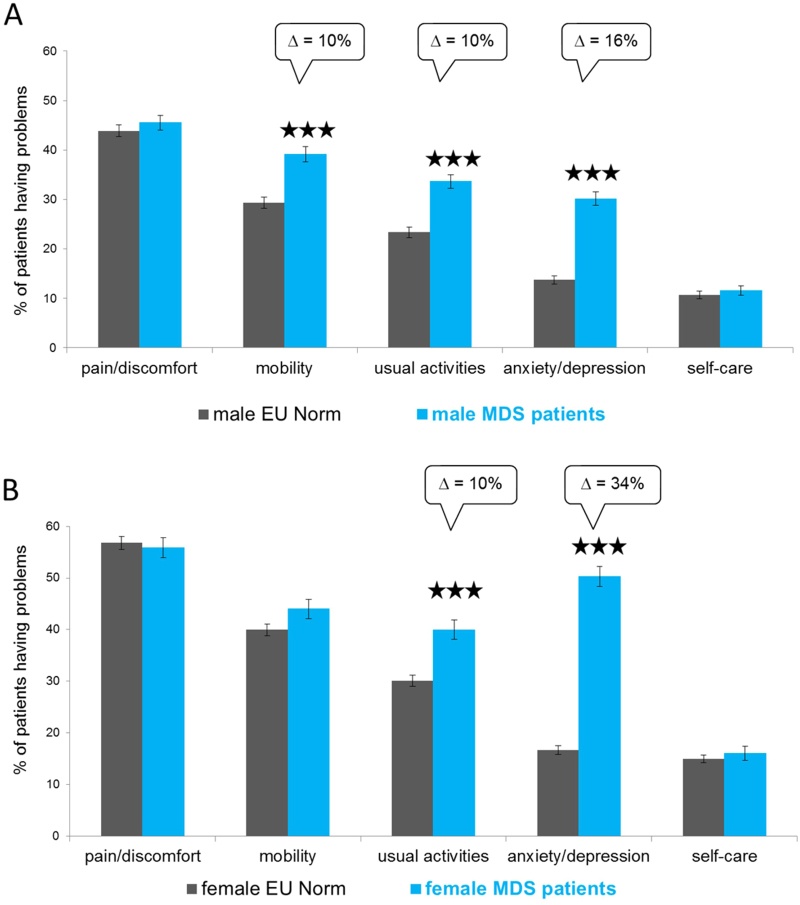
Proportion of moderate/severe problems in male (**a**) and female (**b**) patients with MDS (blue bars) as compared to European age- and sex-matched standard population (dark grey). Standard errors indicated as lines. Differences (∆) of patients with MDS to sex-matched reference group shown when significant (*** *p* < 0.001; **p < 0.01; *p < 0.05; as assessed by Wilcoxon signed rank tests)

**Fig. 2:**
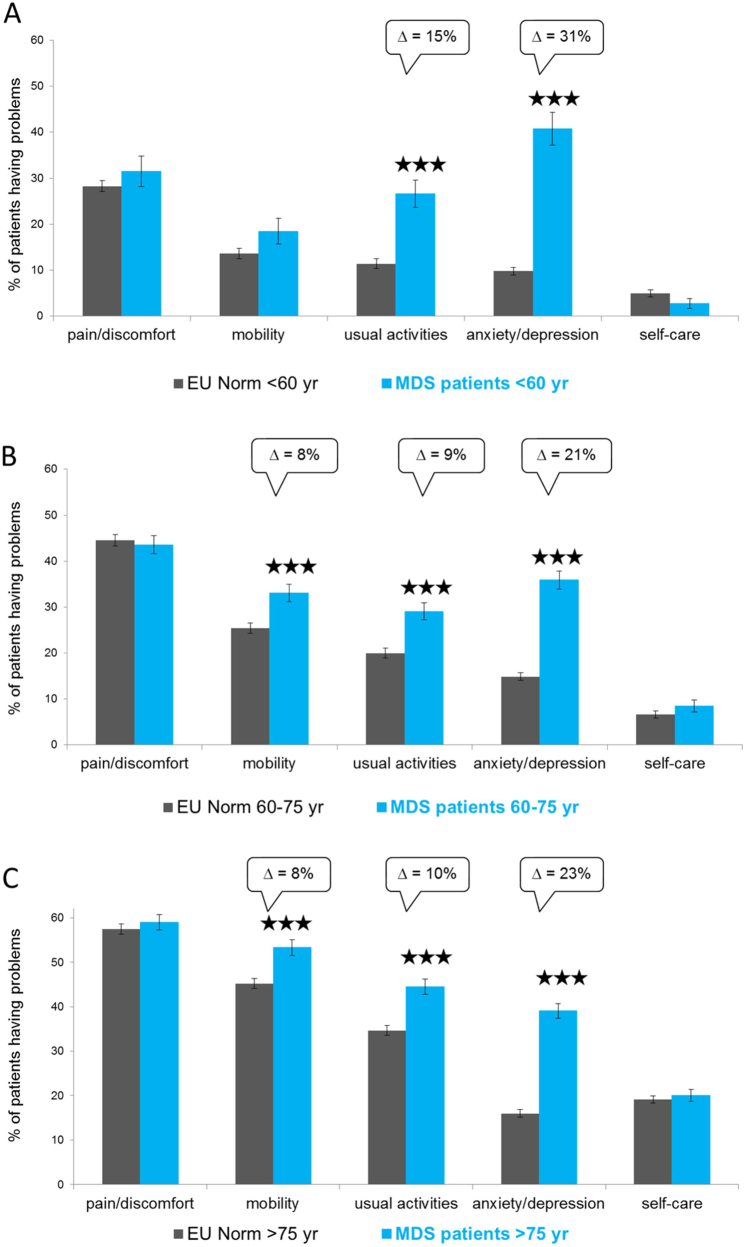
Proportion of moderate/severe problems by age group (<60 (**a**), 60–75 (**b**), or >75 (**c**) years old) in patients with MDS (blue bars) as compared to European age- and sex-matched standard population (dark grey). Standard errors indicated as lines. Differences (∆) of patients with MDS to sex-matched reference group shown when significant (***p < 0.001; **p < 0.01; *p < 0.05; as assessed by Wilcoxon signed rank tests)

Analyses stratified by sex and age depicted most pronounced differences in the dimensions anxiety/depression, and usual activities, in all age groups, and in both sexes (*p* < 0.001). Compared to peers, prevalence of problems in anxiety/depression was most prominent in female (16.7% vs. 50.3%; Fig. 1b) and in younger patients (9.8% vs. 40.8%, *p* < 0.001; Fig. 2a). Restrictions in mobility were most pronounced in male (Fig. 1a) and in older patients (60+ years; *p* < 0.01; Fig. 2c). The dimensions self-care and pain/discomfort were not different between the cohorts (Table 2; Figs. 1 and 2). Differences in EQ-5D index were most pronounced in younger MDS patients (<60 years). EQ-VAS was more often diminished at advanced age (75+ years) as compared to peers (*p* < 0.001; Table 2). These differences fulfilled the criteria of a MID.

## Discussion

This prospective cohort observational study adds substantial information on the prevalence and clustering of restrictions in HRQoL in lower-risk patients with MDS at diagnosis. In a cross-sectional analysis, we observed profound restrictions in distinct dimensions of the EQ-5D when compared with European reference populations. Moreover, we identified demographic and clinical factors, which are associated with restrictions in HRQoL.

### Prevalence of restrictions in HRQoL in MDS at initial diagnosis / Factors associated with decreased HRQoL

Data on symptom burden in lower-risk MDS at initial presentation are rare, and limited by small sample size [16, 17], selection bias [7, 16, 17], and analyses performed later after initial diagnosis [7, 11, 16, 18, 19]. In addition, most studies have included patients with higher risk MDS [9–11, 16, 18–20], AML [10, 11] or CMML [11, 16], which precludes precise interpretation. The strength of our study is the large number of observations at initial diagnosis and the parallel analysis of the different parameters of the validated score EQ-5D including EQ-5D VAS, EQ-5D index as well as the different EQ-5D dimensions in a homogenous cohort of lower-risk patients. This is the first report to present details on restrictions in the distinct domains of EQ-5D in MDS, which reveals huge differences in HRQoL-profile in daily activities. These findings are particularly relevant, as studies from the literature reported exclusively EQ-5D summary scores and EQ-5D VAS [16, 20], but lacked a presentation of EQ-5D daily activities.

Our study shows a pronounced symptom burden in many patients with MDS, predominantly in the dimensions pain/discomfort, mobility, anxiety/depression, and usual activities. Moreover, a clustering of symptoms in distinct subgroups of patients is revealed. The low percentage of self-reported problems in the dimension self-care, particularly in elderly is remarkable. This phenomenon has been observed across different cancer types [28] and may be explained by focusing on “washing and dressing” in the definition of self-care, whereas functional capacities like “work, housework, family or leisure activities” are assessed in the dimension “usual activities”.

We demonstrated that advanced age, pronounced co-morbidities, low Hb-levels, RBCT need, and female sex were significantly associated both with a decreased EQ-5D index, and decreased EQ-VAS after adjustment for co-variables. These observations extend data from the literature [7, 8, 18, 20] and define cohorts of patients which are at high risk of decreased HRQoL. Hb levels [7, 18, 20] and transfusion dependence [20] are important predictors of HRQoL, both in this study and in the literature. Effective treatment for anaemia and reduction of transfusion need might thus contribute to improvement and maintenance of HRQoL [17]. Future studies will focus on the prediction of deterioration of HRQoL, and focus on early prevention.

A relevant aspect of our work is the significant difference in symptom burden in patients with MDS as compared to age- and sex matched European reference populations. Thus, dissection of features which are MDS-specific from symptoms which are present in matched general populations is possible. This study reveals an incremental symptom burden in MDS characterized by pronounced age- and sex-dependent differences in the distinct EQ-5D dimensions. Both young and old patients suffer from troublesome MDS-related symptoms. Data from the literature are rare and have been characterized by a small sample size and were restricted to one country [16, 17]. The study of Hellstrom evaluated HRQoL at later time points after diagnosis, and was focused on selecting anaemic patients with a high probability for response to ESAs for a clinical study [17]. The study of Jansen [16] reported exclusively EQ-5D VAS but lacked a presentation of EQ-5D daily activities for which we show strong differences. Moreover, patients in Jansen's study were entered at variable time points after diagnosis, and included patients with higher risk MDS and CMML [16].

The high prevalence of anxiety/depression and of limitations in usual activities is more pronounced in women in our study. These observations form the basis to appreciate the relevance of MDS on individual health in a given patient and the opportunity to assist health care providers in managing the relevant symptoms [8]. Thus, patient-centred care will be improved by special attention to patient subgroups [29, 30]. The finding of the difference of depression between our MDS patients and the general population is corroborated by similar evidence in other haematologic conditions. For example, Efficace et al. [31] observed that depression was one of the most impaired psychological domains in a sample of chronic myeloid leukaemia patients as compared to their peers in the general population; and, similar to our findings, this impairment was most pronounced in female patients. In agreement with other studies [8, 32, 33], differences by gender were observed with lower HRQoL being more pronounced in females. Although the discussion of causes of disparity in gender-based distribution is beyond the scope of this manuscript, gender-specific evaluations and interventions should be discussed or suggested in patients with MDS.

The relevance of anxiety/depression in patients with MDS is supported by the fact that 9.5% of EU-MDS patients receive antidepressants at baseline [21], and that impairments in depression screening by geriatric depression scale (GDS) are observed in 24% of patients with MDS [34]. Likewise “emotional health” and “uncertainty/sense of control” have been highly ranked by patients and caregivers in a recent study [35]. To address the individual needs of patients with MDS, the novel, disease specific score for MDS, QUALMS [18, 35], is currently applied and validated in the EUMDS-cohort. Our study also confirms that age- and sex-dependent baseline values in HRQoL should be considered when interpreting the results of clinical studies in MDS that use HRQoL as an endpoint, as suggested recently [4, 8].

*Strengths* of this work are the large number of observations, the well-defined inclusion criteria in a non-interventional registry, the enclosure of newly diagnosed MDS patients within 100 days of the date of the diagnostic bone marrow aspirate, and the parallel analysis of the different parameters of the validated generic score EQ-5D [21]. Based on the use of a generic questionnaire, comparisons with reference populations are possible.

*Limitations*: Disease-specific scores may more accurately reflect the spectrum in a given disease. To address this aspect, the MDS-specific score QUALMS has been developed recently [18, 35]. QUALMS has been integrated in EUMDS in a recently amended version of the protocol. Based on objectives of this study and the EUMDS registry, analyses have been restricted to IPSS lower-risk MDS. Therefore, this study does not allow conclusions on MDS in general. However, the recently introduced new protocol of the registry will register all subtypes of MDS. Other aspects of HRQoL, which might be relevant for the outcome of patients, e.g., the deterioration of HRQoL over time, have not yet been analyzed. These investigations are currently performed in several studies focusing on the impact of specific interventions on HRQoL.

### In summary

This is the first study to analyze prospectively the PRO HRQoL in IPSS lower-risk MDS at diagnosis, and to compare patients with MDS with age- and sex-matched healthy populations. Patients experience profound age- and sex-dependent restrictions in different HRQoL dimensions. Distinct demographic and disease parameters are associated with reduced HRQoL. These observations should form the basis for individualized treatment directed at relief of distinct symptoms. In addition, these results may provide a benchmark in the evaluation of new interventional options aimed at improving HRQoL outcomes.

Supplementary Materials is available at Leukaemia (www.nature.com/leu) providing additional information regarding (i) EQ-5D index and EVS; (ii) on the comparison of patients with MDS and the reference population; (iii) on multivariate analysis; and (iiii) on minimally important difference (MID).

## Electronic supplementary material

Additional information on EQ-5D, EVS, reference population and MID
