# Supplementary material for: Health-related quality of life in lower-risk MDS patients compared with age- and sex-matched reference populations: a European LeukemiaNet study
Source: Leukemia. 2018 Mar 6;32(6):1380–92. doi: 10.1038/s41375-018-0089-x (PMC5990524; doi:10.1038/s41375-018-0089-x)
Supplement: Supplementary file 1 — Additional information on EQ-5D, EVS, reference population and MID [file 41375_2018_89_MOESM1_ESM.pdf]

Health-related quality of life in lower-risk MDS patients compared with age- and sex-matched reference populations: a European LeukemiaNet study

Reinhard Stauder* 1, Ge Yu2, Karin A. Koinig1, Tim Bagguley2, Pierre Fenaux3, Argiris Symeonidis4, Guillermo Sanz5, Jaroslav Cermak6, Moshe Mittelman7, Eva Hellström-Lindberg8, Saskia Langemeijer9, Mette Skov Holm10, Krzysztof Mądry11, Luca Malcovati12, Aurelia Tatic13, Ulrich Germing14, Aleksandar Savic15, Corine van Marrewijk9, Agnès Guerci-Bresler16, Elisa Luño17, Jackie Droste9, Fabio Efficace18, Alex Smith2, David Bowen19, Theo de Witte20

Supplement

Additional information on EQ-5D index and EVS

The EQ-5D index is constructed by applying weights to each of the levels within each dimension in the descriptive system and adding these together for 243 (3^5) possible health states. A set of weights, the EQ-5D European value set (EVS), has been elicited in Western European countries,[1] which we applied to derive the EQ-5D index because the EVS provides a potentially useful platform for this large cross-European comparative study. Another advantage of adopting the EVS is that it can be applied in the six countries in this study (Austria, Czech Republic, Israel, Poland, Romania, and Serbia) for which there is absence of locally appropriate value sets.[2]

Additional information on the comparison of patients with MDS and the reference population

The objective of this paper was to compare the QoL of Patients with MDS with general population with a similar age and gender distribution, rather than the QoL of patients with other diseases including cancer. Population norms can be used as reference data to compare profiles for patients with specific conditions with data for the average person in the general population in a similar age group and/or gender. They aim to be a representative sample of the general population of a country or region and do not make any exclusions based on an individual's comorbidities or previous diseases. EQ-5D population norms are provided from representative national surveys of 20 countries and additional regional surveys conducted. Data collection for the majority of surveys took place during or after 2000, however, some surveys were older with the UK and Swedish national datasets being the earliest from 1993 to 1994, respectively. These surveys differ in sample size and in the method of data collection. The Danish dataset had the largest sample with over 16,000 respondents, while the Greek and the Swedish national surveys had the smallest sample of around 500 respondents.

Additional information on multivariate analysis

Two separate multivariate analyses were performed to assess whether each of the two overall HRQoL measures (EQ-5D index or EQ-VAS) could discriminate between risk groups with different demographic and clinical parameters. Evidence of the ability to discriminate between different risk groups was considered to be provided when there was a significant difference between the HRQoL of different risk groups of patients, where this would be the case for regression coefficients which are significantly different from zero. The significance level was set at $p<0.01$.

Since individual patients (level 1) are nested within centers (level 2) that can also be nested within in countries (level 3), a hierarchical regression model with a three-level nested structure was fitted in the multivariate analyses, in which the individual-level factors are the first level of the analysis, with centres and countries constituting the second and third levels of the analysis, respectively. As such, our models adjust for the unmeasured variation in the confounding characteristics of centres and countries, which may be more or less related to health outcomes.

Additional information on MID

The MID is viewed as the smallest difference in score in the domain of interest that is perceived by patients as beneficial or that would result in a change in treatment.[3] The MID for the EQ-5D index was estimated between -0.011 and 0.140 (mean=0.074) in a literature review of eight longitudinal studies,[4] and was reported as 0.033 in chronic conditions.[5] We aimed for a conservative threshold in this study and inferred that a difference in HRQoL of >0.03 constituted an MID for the EQ-5D index. As we are unaware of any studies which have estimated the MID for the EQ-VAS, we simply assumed a similarity difference of >3.0 on the EQ-VAS constituted an MID.

1. Greiner W, Weijnen T, Nieuwenhuizen M, Oppe S, Badia X, Busschbach J, *et al.* A single European currency for EQ-5D health states. Results from a six-country study. *Eur J Health Econ* 2003 Sep; **4**(3)**:** 222-231.

2. Oppong R, Kaambwa B, Nuttall J, Hood K, Smith RD, Coast J. The impact of using different tariffs to value EQ-5D health state descriptions: an example from a study of acute cough/lower respiratory tract infections in seven countries. *Eur J Health Econ* 2013 Apr; **14**(2)**:** 197-209.

3. Jaeschke R, Singer J, Guyatt GH. Measurement of health status. Ascertaining the minimal clinically important difference. *Control Clin Trials* 1989 Dec; **10**(4)**:** 407-415.

4. Walters SJ, Brazier JE. Comparison of the minimally important difference for two health state utility measures: EQ-5D and SF-6D. *Qual Life Res* 2005 Aug; **14**(6)**:** 1523-1532.

5. Sullivan PW, Lawrence WF, Ghushchyan V. A national catalog of preference-based scores for chronic conditions in the United States. *Med Care* 2005 Jul; **43**(7)**:** 736-749.
